# Iron Absorption in Celiac Disease and Nutraceutical Effect of 7-Hydroxymatairesinol. Mini-Review

**DOI:** 10.3390/molecules25092041

**Published:** 2020-04-27

**Authors:** Isabella Zanella, Giulia Paiardi, Diego Di Lorenzo, Giorgio Biasiotto

**Affiliations:** 1 Department of Molecular and Translational Medicine, University of Brescia Viale Europa 11, 25123 Brescia, Italy; 2 Clinical Chemistry Laboratory, Diagnostic Department, ASST Spedali Civili di Brescia Piazzale Spedali Civili 1, 25123 Brescia, Italy

**Keywords:** 7-Hydroxymatairesinol, iron metabolism, anemia, hepcidin, inflammatory bowel diseases, celiac disease

## Abstract

Anemia is the main extra-gastrointestinal symptom in inflammatory bowel diseases (IBDs). Interleukin-6 (IL-6) and other cytokines are secreted and act in the microenvironment of the small intestine mucous membrane of IBD patients. Iron is essential for multiple cell functions and its homeostasis is regulated by the hepcidin–ferroportin axis. Hepcidin (HEPC) is mainly produced by the liver in response to iron needs but is also an acute phase protein. During inflammation, hepcidin is upregulated by IL-6 and is responsible for iron compartmentalization within cells, in turn causing anemia of inflammation. Tissues other than liver can produce hepcidin in response to inflammatory stimuli, in order to decrease iron efflux at a local level, then acting in an autocrine–paracrine manner. In IBDs and, in particular, in celiac disease (CeD), IL-6 might trigger the expression, upregulation and secretion of hepcidin in the small intestine, reducing iron efflux and exacerbating defective iron absorption. 7-Hydroxymatairesinol (7-HMR) belongs to the family of lignans, polyphenolic compounds produced by plants, and has nutraceutical antioxidant, anti-inflammatory and estrogenic properties. In this mini-review we revise the role of inflammation in IBDs and in particular in CeD, focusing our attention on the close link among inflammation, anemia and iron metabolism. We also briefly describe the anti-inflammatory and estrogenic activity of 7-HMR contained in foods that are often consumed by CeD patients. Finally, considering that HEPC expression is regulated by iron needs, inflammation and estrogens, we explored the hypothesis that 7-HMR consumption could ameliorate anemia in CeD using Caco-2 cells as bowel model. Further studies are needed to verify the regulation pathway through which 7-HMR may interfere with the local production of HEPC in bowel.

## 1. Introduction

The intestine has been defined as the most important lymphoid organ of the human body [1]. Indeed, the gut can suffer from different immune-mediated disorders like inflammatory bowel diseases (IBDs), including Crohn’s disease and ulcerative colitis, primary eosinophilic gastrointestinal disorders and celiac disease (CeD), which can alter both its structure and its functions. In addition to the symptoms related to the gastrointestinal tract, extra-intestinal manifestations often occur in these disorders that complicate the whole clinical picture. Anemia is the most frequent extra-intestine clinical manifestation and contributes to worsening the quality of life of the patients affected by these pathologies [2]. Patients affected by CeD, one the main immune-mediated gastrointestinal disorders, present a high prevalence of anemia, ranging from 12% to 69% [3]. Further, anemia may be the only clinical sign of the disease. Anemia in CeD is mainly caused by malabsorption and iron deficiency, with the contribution of other micronutrient deficiencies, such as folate or vitamin B_12_ shortages [3].

## 2. Celiac Disease and Inflammation

In this mini-review we revise the role of inflammation, particularly in CeD, focusing our attention on the close relationships between inflammation, anemia and iron homeostasis. We also briefly describe the anti-inflammatory and estrogen activity of lignans, polyphenolic compounds, contained in fiber-rich foods, often consumed by CeD patients and widely considered as nutraceuticals. We finally provide preliminary evidence that lignans could ameliorate inflammation and anemia in CeD, showing that lignans could inhibit the local expression of Interleukin-6 (IL-6) and hepcidin, the main regulator of iron homeostasis.

CeD is a permanent sensitivity to gluten that results in a disorder of the small intestine with the involvement of other organs. This disorder derives from the interaction between environmental and genetic factors. The genetic predisposition is linked to the HLA class II haplotype DQ2 or DQ8. HLA DQ proteins are constituted of two subunits, DQ2 and DQ8 receptor isoforms, that bind gliadin peptides with higher affinity than other types. HLA-DQA1 and HLA-DQB1 genes encode for these two subunits and are located on chromosome 6. Among the DQ2 haplotypes, two haplotypes are mainly linked with CeD. Haplotype DQ2.5 characterizes about 95% of CeD patients. The protein specified by α^5^ and β^2^ subunits is encoded by the two alleles DQA1*0501 and DQB1*0201. A small number of CeD patients carry the haplotype DQ2.2, encoded by the gene alleles DQA1*0201 and DQB1*0202. Almost all the remaining CeD patients are characterized by DQ8 haplotypes encoded by the alleles DQA1*03 and DQB1*0302 [4]. Genome-wide association studies (GWAS) have associated 39 genes with CeD heritability, but additional studies are needed to verify its clinical usefulness [5].

Upon ingestion of gluten, the glutamine and proline-rich proteins, gliadin and other prolamins of gluten, are partially digested by gastrointestinal enzymes. In the intestinal mucosa, they are bound and modified by the enzyme transglutaminase 2 (TG2) and may activate the immune innate and adaptive responses. The activated immune system can cross-react against the small bowel tissue, causing an inflammatory reaction that leads to crypt hyperplasia and villous atrophy, thus interfering with the absorption of several nutrients [6,7]. CeD is characterized by variable combinations of elevated titers of celiac-specific autoantibodies, inflammatory enteropathy with various degrees of severity and a wide range of gastrointestinal and extraintestinal symptoms [8,9]. The increase of different autoantibodies has been associated with the worsening of villus atrophy, described by the use of the Marsh classification [10]. The peptides reach the intestinal mucosa by abnormal transcellular and paracellular transport. Recent studies have evidenced that gluten components, such as gliadin and other prolamins, can bind the C-X-C motif chemokine receptor 3 (CXCR3), which can induce the increase of intestinal zonulin and disassemble intercellular tight junctions of the intestinal mucosa, thus altering gut paracellular permeability and substantially contributing to the pathogenesis of CeD [11,12,13]. The transcellular transport of gliadin is induced by Interferon gamma (IFN-γ) and is mediated by the Transferrin Receptor 1 (TFR1) that binds the secretory IgA-gliadin complexes. Indeed, TFR1 is overexpressed in the apical pole of enterocytes in active CeD and allows the retrotranscytosis of these complexes without lysosomal degradation. This mechanism allows the translocation of gliadin peptides in the gut mucosa [14].

The binding of the gluten peptides on HLA-DQ2 and HLA-DQ8 proteins raises an innate and adaptive immune response in CeD patients [15]. The presentation of the complex formed by HLA isomers and undigested peptides by antigen-presenting cells (APC) to CD4+T cells located in the lamina propria triggers the adaptive immune response with the production of IFN-γ and other pro-inflammatory cytokines [16]. These cytokines trigger T helper-1 response with production of interleukin-15 (IL-15) and interleukin-21 (IL-21) and consequent activation of cytotoxic CD8^+^ intraepithelial lymphocytes. These cells contribute to intestinal damage and inflammation [17]. IL-15 and IL-21 stimulate T-helper 2 cells that are responsible for the differentiation of B-lymphocytes, which produce anti-TG2 and anti-gliadin antibodies characteristic of active CeD [16,18]. Under the influence of IL-15, epithelial cells express the surface glycoproteins MHC class I polypeptide-related sequence A (MICA) and MHC class I polypeptide-related sequence B (MICB) produced by stress-activated genes. These glycoproteins are recognized by the receptors NKG2D and CD9/NKG2A of intraepithelial natural killer T-lymphocytes, which induce apoptotic death of enterocytes [19]. A common feature of CeD and many organ-specific autoimmune diseases is the central role of T and B cells in causing tissue destruction [6]. CeD is characterized by a strong and pathogenic response of CD4^+^ T-cells against deaminated gluten peptides and at the same time a B-cell mediated response against the same peptides and the self-protein TG2 [20]. The production of autoantibodies against the complex gluten peptides and TG2-gluten by B-cells corresponds to the clinical onset of the disease. Recent evidence suggests that B-cells may further contribute as antigen-presenting cells that can activate the pathogenic gluten-specific T cells, thus initiating autoimmune conditions [21]. The increase of gliadin-specific CD4^+^ T cells, in addition to the secretion of proinflammatory cytokines such as IFN-γ and interleukin 21 (IL-21), could contribute to promoting the triggering of intraepithelial lymphocytes (IELs) [22,23]. IELs are mainly composed of several types of T-cells CD3+ and CD8+T lymphocytes and, fewer in number, CD4+T lymphocytes [24]. The production of cytokines is a fundamental trigger for the inflammation of the gut mucosa and various mechanisms of production and different immune cell contributions are described in the literature.

T-cell recognition of gluten antigens is a critical step and the subset of pro-inflammatory T helper cells involved in the immune response of small intestinal mucosa (Th17 subset) is characterized by the production of interleukin 17 (IL-17). The increase of interleukin 1 beta (IL-1β), interleukin 6 (IL-6) and IL-17 has been reported in biopsy specimens from patients with untreated CeD [25].

Monocytes and dendritic cells have also been considered as implicated in CeD. These cells are unique in that they can directly react to gluten as well as support inflammatory T helper cells that are gluten-responsive. When dendritic cells are cultured with gliadin fragments derived from proteolytic digestion, they produce the inflammatory cytokines IL-6, interleukin 8 (IL-8) and tumor necrosis factor alpha (TNFα) [26]. When exposed to interleukin 15 (IL-15) and proteolytic fragments of gliadin, monocytes can also activate an adaptive immune response to wheat by producing IL-1β, IL-6, IL-15, interleukin 23 (IL-23), TNFα and chemokine (C-C motif) ligand 20 (CCL20). Furthermore, the exposure to gliadin induces the Th17 response mentioned above and a Th1 response mediated by CD4^+^ T cells and characterized by IFNγ production [27].

After the contact with gluten, monocytes are heavily involved in the alteration of the intestinal mucosa barrier. In fact, they produce inflammatory cytokines and interact with epithelial cells [28,29,30]. Monocytes, coming from blood flow, infiltrate the lamina propria and differentiate into macrophages that produce inflammatory cytokines, such as TNF-α and IL-8, after coming into contact with gluten [31,32]. When monocytes, isolated from peripheral blood of CeD patients, are co-cultured with intestinal epithelial cells, they show the ability to modify the cell layer, generating barrier functional defects and tight junction alterations by means of cytokine production, mainly IL-6 and monocyte chemoattractant protein 1 (MCP1) [33]. A study with intestinal biopsy cultures obtained from celiac disease patients showed that regulatory T cells exist in the mucosa of untreated celiac disease patients, but they are dysfunctional due to the overexpression of IL-15 derived from epithelial cells [34].

**Figure 1:**
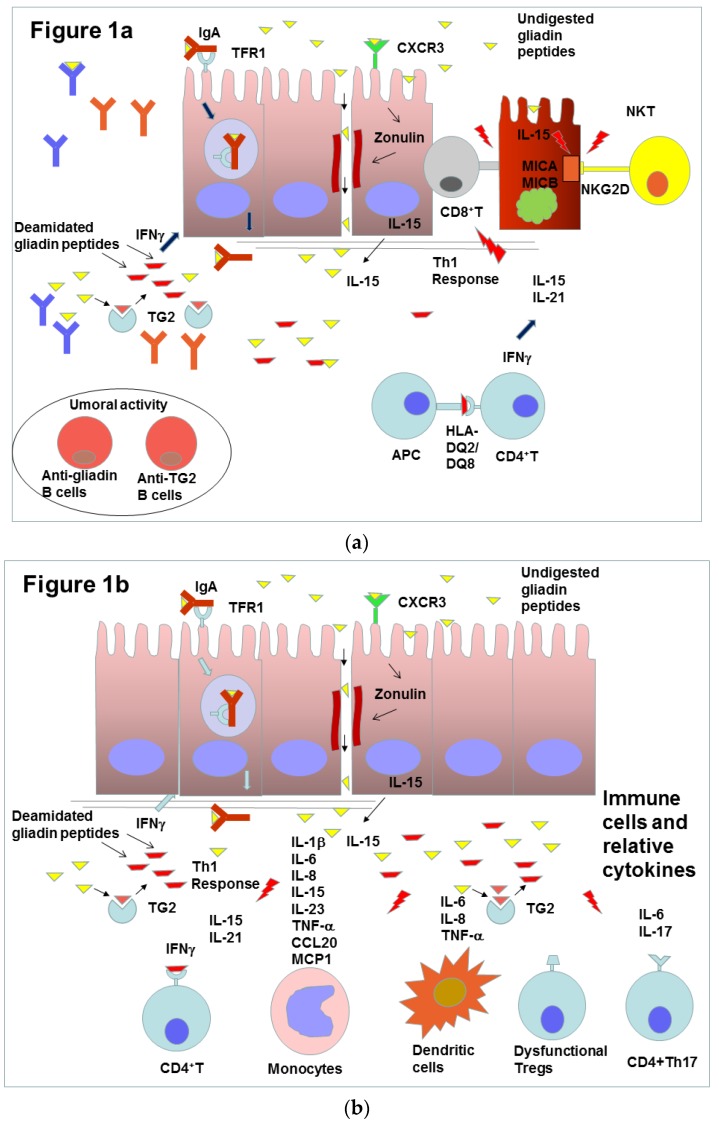
(**a**) Schematic representation of the effects of gliadin peptides on gut mucosa and immune cells. Gliadin peptides reach lamina propria, altering paracellular permeability by inducing zonulin expression or through transcytosis of gliadin peptides-IgA complexes mediated by transferrin receptor 1 (TFR1). Gliadin peptides are deaminated by the enzyme transglutaminase 2 (TG2). The deaminated peptides are presented bound to HLA-DQ2/DQ8 by APC to CD4+T cells and induce Th1 response, activating IELs such as CD8+Tcells, and Th2 response, activating B cells that produce specific antibodies against gliadin peptides and TG2. IL-15 stresses the epithelial cells to produce the surface glycoproteins MICA and MICB, which are recognized by the receptors NKG2D and CD9/ NKG2A of NKT-lymphocyte (NKT). When NKT receptors bind these molecules, they cause apoptotic death of the enterocytes. Figure (**b**) shows different types of leukocytes and in particular monocytes, dendritic cells and CD4+Th17 cells, which produce cytokines that contribute to enhance gut inflammation.

Gluten proteins can generate toxic peptides when digested by intestinal enzymes. In particular α and γ gliadin produce the more toxic and immunogenic fragments. Among the peptides generated by α2-gliadin digestion, two have been mainly studied: the 25-mer p31–55, that can generate smaller fragments such as p31–43, and the 33-mer p-57–89 [5]. The p31–43 peptide can stimulate immune innate responses in CeD and induces the expression of cell surface IL-15 in human intestinal epithelial Caco-2 cells, thereby stimulating the proliferation of T cells [35]. The induction of an epithelial pro-inflammatory phenotype may alter the first mucosal defense against ‘toxic’ agents, leading to a wide perturbation of the regulatory mechanisms at the mucosal surface. The epithelial uptake of some toxic gliadin peptides, in particular p31–43, induces an intracellular pro-oxidative environment, favoring the production of autoantibodies specific for the endogenous enzyme TG2 and leading to an innate immune response. Stimulation of Caco-2 cells with proteolytic fragments of gliadin induces the secretion of two key pro-inflammatory cytokines, IL-6 and IL-8 [36]. The treatment with gastric-pancreatic enzymes and further digestion by intestinal brush border enzymes of both the recombinant α2-gliadin and the whole gliadin, extracted from a common wheat cultivar, have evidenced the generation of a new peptide, p31–55, that contains the encrypted sequence p31–43. Although the peptide p31–43 is a consolidated tool to study the toxicity of gliadin derivatives, it is not produced and released by enzymatic hydrolysis [37]. The permeability of the peptide p31–55 through the epithelial cell layer of confluent Caco-2 cells has been demonstrated and it is increased in the presence of whole digested gliadin extracts from a common wheat cultivar. These findings have evidenced that this peptide is resistant to gastro-intestinal digestion and is permeable in the proposed in vitro model; therefore, it may play a role in the activation of innate immunity [38]. The peptide p31–43 activates IFN-α, a molecule implicated in the innate immune response, which works together with a viral ligand activating the toll-like receptor 7 (TLR7) pathway by modifying endocytic trafficking. Therefore, there is evidence that dietary peptides and viral infections together could potentiate innate immunity, by inducing the autoimmune response in CeD [39] (Figure 1).

Considering that anemia is one of the most significant symptoms in CeD, it must be taken into account that inflammation and iron metabolism are closely related. Pro-inflammatory cytokines such as IL-6 and IL-1β can induce hepcidin expression with the consequent iron compartmentalization and decrease of iron absorption [40]. This condition may determine iron-restricted erythropoiesis and anemia of inflammation, worsening the effect of malabsorption.

## 3. Iron Homeostasis and Hepcidin

Iron is essential for multiple cell functions, but it can also be potentially deleterious because of its ability to generate free oxygen radicals. Due to the lack of a mechanism that actively eliminates iron, in mammals its systemic balance is maintained by controlling its intestinal absorption and continuous cellular recycling. The daily losses of iron are small (1–2 mg in adults), representing about 0.1% of the 3–4 g of total body iron, and are due to epithelial exfoliation. To maintain iron balance, its losses must be replaced by food intake. Most iron in the body is contained in red cell hemoglobin. In fact, the iron content of blood is 0.5 mg per milliliter, reaching about 2–3 g in total. In the human body, the need to generate 200 billion erythrocytes per day requires 20 mg of iron that mainly comes from hemocatherisis sites in bone marrow [41]. When the iron level decreases in the blood, its absorption is increased by intestinal enterocytes through transporters of the luminal side. These cells increase the expression of the channel transporter divalent metal transporter 1 (DMT1) and of the ferrireductase protein duodenal membrane associated cytochrome B (DcytB). DcytB reduces the food iron from Fe^3+^ to Fe^2+^, that is soluble and can enter the enterocytes by means of DMT1 [42]. Iron complexed in the heme group is absorbed by means of a dedicated transporter that is however not yet identified. Iron, entered in the enterocyte cytoplasm, forms the labile iron pool (LIP), transiently complexed with chaperone molecules and reaches the basolateral membrane to be released in the blood circulation. This egress from the enterocytes is mediated by ferroportin, (FPN also named SCL40A1), the only known iron exporter. Iron is then oxidized to Fe^3+^ by the copper-containing enzyme hephaestin (HEPH), bound to and transported by transferrin (TF) circulating in the blood, made available for cellular needs and recycled in few hours. The continuous recycle moves about 20 mg/day of iron bonded to transferrin mainly from sites of hemocathereris to sites of erythropoiesis. This transport is relatively fast and occurs within hours. Therefore, the mean of the iron that transits in blood is about 2–3 mg. About 1 g iron is primarily stored in hepatocytes and liver and spleen macrophages. Stored amounts are lower in women during reproductive age, in part due to blood losses from menstruation and parturition. In hepatocytes and macrophages, iron is stored in cytoplasmic ferritin (FT), the iron storage protein described below, and is readily mobilized during high iron demand [41,43].

TF is a glycoprotein with two iron binding sites. TF binds iron in its oxidized form, preventing iron-mediated free radical formation and is about 30% saturated in normal conditions. The copper enzyme ceruloplasmin (CP), circulating in the blood flow, can oxidize iron to help TF iron binding, contributing to controlling oxidative stress. Iron bound to TF is the main source for the needs of all cells and in particular for erythroid cell precursors. The complex Fe-TF binds TFR1 and is internalized by endocytosis. The acidic pH of early endosomes allows iron release, while TF remains linked to TFR1. The complex TF-TFR1 returns to the cell surface, where it dissociates, enabling the release of free TF in the blood flow. Inside the endosome, the reductase, six-transmembrane epithelial antigen of prostate 3 (STEAP3) reduces iron to Fe^2+^, that is transported in the cytosol by DMT1, contributing to LIP formation [44]. The LIP can take different paths: storage, utilization or cell release. The majority of LIP is used by mitochondria for Fe-S clusters and heme synthesis [45]. Like in enterocytes, the export of iron is performed by FPN in all other cells. Iron that is not used or exported is stored within the nanocage of FT, a cytoplasmic heteropolymeric protein constituted by 24 subunits. The heteropolymer is composed of different ratios of the light chain ferritin (FTL) and heavy chain ferritin (FTH) and can store up to 4500 iron atoms [46].

Two proteins named iron regulatory proteins (IRPs) control iron homeostasis of the cells. These proteins, named IRP1 and IRP2, exercise their control in a post-transcriptional way, interacting with a secondary stem loop structure, named iron responsive elements (IRE) and located in the untranslated regions (UTRs) of iron-related protein mRNAs. When iron concentration in the cell is low, IRPs bind IRE; if iron increases, these proteins leave the IRE, IRP1 binds a Fe-S cluster and works as a cytoplasmic aconitase, while IRP2 is degraded. IRP binding activity is high in iron deficiency and hypoxia states and is suppressed by iron and oxygen increase. IRPs, using this mechanism, orchestrate the coordinated expression of iron importers (TFR1 and DMT1), storage (FT) and export (FPN) proteins and very quickly respond to changes in cellular iron concentrations [47].

Hepcidin (HEPC), a 25 amino acid peptide mainly produced by hepatocytes of the liver, is the hormone that controls systemic iron metabolism. HEPC inhibits iron entry into the plasma compartment through the three main sources of iron entry: dietary absorption by enterocytes in the duodenum, release of recycled iron from macrophages and release of stored iron from hepatocytes [48]. HEPC inhibits cellular iron efflux by directly binding to FPN, inducing its conformational change and triggering the endocytosis of both molecules, with their consequent lysosomal degradation [49]. A different mechanism has been evidenced in duodenal cells, where hepcidin binding of FPN determines the decrease of iron transport without the internalization and degradation of the HEPC-FPN complex. In these cells, the lowering of iron absorption is indeed due to the decrease of DMT1 expression rather than a decrease of FPN [50,51,52].

HEPC synthesis by hepatocytes is transcriptionally regulated by iron, through the bone morphogenetic protein (BMPs) signaling pathway that creates a feedback mechanism that prevents iron overload [47,53]. Recent studies, mainly in animal models, have demonstrated the expression of HEPC in tissues or cells other than liver and hepatocytes such as heart, kidney, macrophages, stomach, adipose tissue, pancreas, brain, lung, placenta, retina, skin and bowel [54,55,56,57]. In all these districts, the basal expression of HEPC is lower than that in the liver and it is thought to play a role in the local regulation of iron homeostasis in an autocrine and paracrine manner [55,56,57,58]. 

It has been reported in the literature that estrogens can regulate HEPC expression. 17β-estradiol (E2) downregulates hepcidin expression through an estrogen responsive element (ERE) half site in HuH7 and HepG2a cell lines used as cellular models of liver. This site is located upstream of the transcription start site in the HEPC gene (HAMP) promoter between -2474 and -2462 [59]. Another ERE has been found at −1244 to −1232, able to induce decrease of HAMP transcription in the hepatocarcinoma cell line SMMC-7721 [60]. Estrogens are reported to change iron metabolism, downregulating hepcidin also in MCF7 cells [61]. The same effect has been found in patients during in vitro fertilization with endogenous estrogens at high levels when compared with suppressed patients using a gonadotropin releasing hormone agonist buserelin [62]. This and other evidence suggests the existence of an iron–estrogen axis that protects premenopausal women from iron deficiency and that could be dysregulated in some diseases [63].

**Figure 2:**
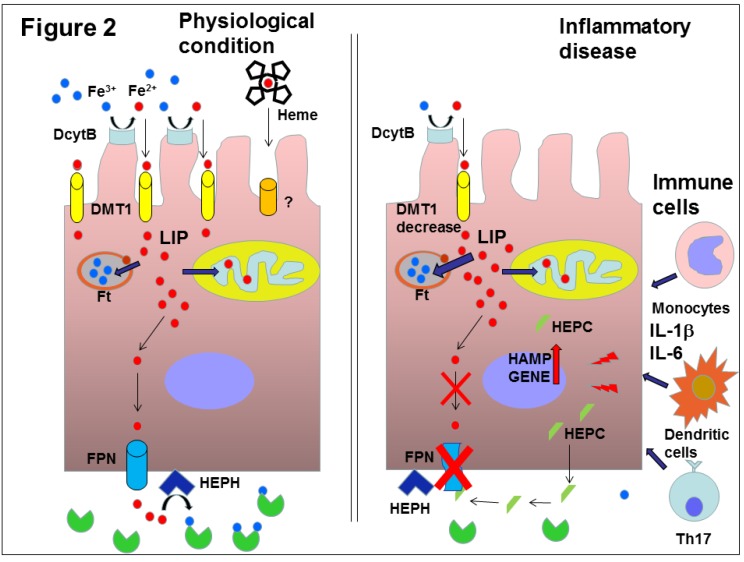
In physiological conditions, DcytB reduces the intestinal iron from Fe^3+^ to Fe^2+^ that can enter the enterocytes by means of DMT1. Iron, complexed in the heme group, is absorbed through an as yet unknown transporter. Iron enters in the enterocyte and constitutes the labile iron pool (LIP) that reaches the basolateral membrane to be released in the blood circulation by ferroportin (FPN). Iron is then oxidized to Fe^3+^ by hephestin (HEPH) and is bound and transported by transferrin (TF) circulating in the blood to satisfy body need. Under inflammation, hepcidin (HEPC) expression is upregulated through cytokines produced by IELs, such as interleukine-6 (IL-6) and interleukine-1beta (IL-1β). In enterocytes, the expression of HEPC could play a role in local iron homeostasis regulation, in an autocrine/paracrine manner. HEPC might induce FPN degradation or inactivation, decreasing iron efflux. The consequent increase of cellular iron might determine the diminution of DMT1 expression to decrease iron absorption. Therefore, this mechanism, caused by local inflammation, might exacerbate defective iron absorption due to reduced absorptive surface area in the intestine of patients presenting with anemia.

During inflammation, the HAMP is upregulated by IL-6, IL-1β and other cytokines as well as by lipopolysaccharide (LPS) through the Janus kinase 2 (JAK2)/signal transducer and activator of transcription 3 (STAT3) signaling pathway [64]. This molecular mechanism increases HAMP transcription and HEPC blood concentrations during infections and systemic inflammatory diseases. The rise of HEPC causes hypoferremia that develops very rapidly during infections or inflammatory diseases. Although the efficacy of this mechanism in host defense against specific microbes is not fully demonstrated, the most likely hypothesis is that this response could limit the growth of iron-dependent extracellular microbes [40]. As for other defense responses against pathogens, there is a price to be paid. Inflammation determines iron sequestration and hypoferremia through blood HEPC increase. This condition limits the availability of iron needed to produce erythrocytes, and causes the onset of anemia of inflammation (also known as anemia of chronic disease) [43,65] (Figure 2). Chronic inflammatory disorders including infections, rheumatologic disorders and IBDs are indeed associated with hypoferremia and anemia. This type of anemia is mild or moderate, mainly normocytic and resistant to iron therapy, but sometimes it may manifest as hypochromic and microcytic anemia [65]. Several studies in the literature show blood HEPC increases in patients affected by these disorders [65,66].

The genetic background of CeD patients could influence iron metabolism and the ability to resolve anemia after diagnosis and compliance with gluten free diet. Two of these modifier genes are HFE (H= high FE = Fe, iron homestasis regulator) related with type 1 hemochromatosis [67] and TMPRSS6 (transmembrane serine protease 6) coding for the matriptase 2 and causative of iron refractory iron deficiency anemia (IRIDA) [68]. The frequencies of the more common HFE mutations H63D and C282Y have been studied in a cohort of Italian CeD patients, but no significant differences in comparison to control subjects were found. Histological damages seem to be mainly responsible for alterations in iron metabolism of these patients, but females carrying the C282Y variant seemed to recover from anemia faster than other patients after a gluten free diet [69]. The TMPRSS6 A736V variant has been found more frequently in CeD patients than in controls. However, when CeD patients on a gluten free diet were stratified in affected or not affected by persistent iron deficiency anemia, no differences have been found between the two cohorts [70]. The effects of the HFE mutations H63D and C282Y and of the TMPRSS6 A736V variant were further studied in two cohorts of CeD patients with and without persistent iron deficiency anemia after diagnosis and after being on a gluten free diet for one year. HFE mutations showed a protective role against iron deficiency anemia, while the presence of the TMPRSS6 variant could help to predict oral iron response in anemic patients. The cohort of patients with iron deficiency anemia had lower HEPC levels in the blood than the group without anemia. The level of HEPC is modulated by different genotypes and, in both cohorts of patients, decreased during the follow up, likely for inflammation decrease. [71,72].

Numerous recent studies have focused their attention on the possibility of pharmacologically modulating HEPC expression [65], with the intent to reduce anemia in chronic diseases. Among these molecules, some natural compounds could be used as nutraceuticals [73,74,75,76,77,78].

## 4. 7-Hydroxymatairesinol

Lignans are polyphenolic chemicals produced as secondary metabolites in plants where they are mostly found as aglycones, oligomers and glycosides. Their structure is determined by the union of two cinnamic acid residues or by their biogenetic equivalents [79,80]. They occur in the whole plant kingdom and can also be found in fiber-rich foods, typically regarded as healthy foods. Dietary lignans are broadly available and particularly concentrated in oilseeds (especially in flaxseed and sesame), in cereal grains (e.g., wheat and rye bran) and nuts, Brassica species, legumes, berries, and in many plant-related beverages (tea, coffee, wine) [80,81]. Lignans are transformed, completely or in part, by gut microbiota into the enterolignans enterodiol (END) and enterolactone (ENL). A high intake of edible plants containing lignans has been demonstrated to reduce the incidence of certain chronic diseases in human and animal models [80,82]. Sesame and flaxseed are lignan-rich foods which, in mice fed a high-fat diet, improve weight, metabolic parameters, lipid profiles and restore the expression level of TNFα down to the level found in mice fed a low-fat diet. These data showed an efficient amelioration of diet-induced inflammation [83]. Other evidence indicates that plant lignans may be potent anti-inflammatory agents as they show higher antioxidant activity than enterolignans [84]. Moreover, they have been reported to exhibit anti-inflammatory activity through the inhibition of mitogen-activated protein kinases (MAPKs) phosphorylation [85].

7-Hydroxymatairesinol (7-HMR) is a plant lignan characterized by a structure of dibenzylbutyrolactone derived from matairesinol. This lignan is contained in knots of Norway spruce or European spruce trees (*Picea Abies*) and in different concentrations in edible plants [86,87]. Different methods have been developed in the last years to isolate pure 7-HMR from knots and today this lignan can be purified on a Kg-scale [88].

Among plant foods, 7-HMR is present in oilseeds and cereals such as wheat, triticale and oat, mainly in its bran fraction [86]. In particular, rye, millet, maize bran and amaranth are gluten free cereals and are often components of bakery products normally consumed by CeD patients (Table 1). This lignan is converted by intestinal microflora into its main metabolite ENL and other minor metabolites, like END, allo-7-HMR and unmodified 7-HMR [89].

7-HMR has anticancer, antioxidant, anti-inflammatory, immunomodulatory and estrogenic properties. Anticancer and chemoprotective properties have been shown in various animal models. Uterine cancer and mammary tumor models were examined in rats [89,90], and intestinal tumor and prostate cancer models were studied in mice [91,92]. 7-HMR antioxidant properties have been shown in endothelial cells and various tumor models [85,89,93,94].

In Wistar rat animal model, high doses of 7-HMR such as 160, 640 and 2400 mg/kg were administered to verify potential toxicity. All doses were well tolerated with an improvement of blood lipid profile [95]. The toxicity was tested in the same model, also to evaluate prenatal development of fetus. The doses administered with food were 0.14–0.18, 0.46–0.74, and 1.19–2.93 g/kg. The data obtained from this study evidenced no effect on reproductive performance and no anomalies in the fetus development [96]. In a different strain of Donryu rat as model of uterine carcinogenesis, 7-HMR was administered in food at doses of 200 mg/kg and 600 mg/kg, obtaining a significant reduction of incidence of this type of tumor [90]. In Sprague–Dawley rats affected by induced mammary carcinoma, 7-HMR was administered via diet at daily dose of 4.7 mg/Kg. The results obtained in this study were contradictory. In general, tumor volume and tumor growth decreased, the incidence of well-differentiated adenocarcinoma decreased, while the poorly differentiated adenocarcinoma and atrophic type increased [97]. To evaluate the potential to be a nutraceutical, 7-HMR was administered in a clinical study to analyze healthy postmenopausal women. When introduced in food at dose of 72 mg/day, 7-HMR showed no toxicity and evidenced a significant improve of hot flash frequency [98].

There is evidence of significant anti-inflammatory effects of 7-HMR and its major stereoisomer, 7-hydroxymatairesinol 2 (7-HMR2), through the inhibition of the nuclear factor-kB (NF-kB) and the extracellular signal regulated kinases (ERK) phosphorylation [94]. 7-HMR at a dose of 3 mg/kg has been shown to improve weight and metabolism of fat and sugars in a study on male mice, used as a model of metabolic syndrome, and these properties were confirmed in the 3T3-L1 cell line used as adipogenesis model. Furthermore, this lignan showed anti-inflammatory effects, causing downregulation of TNFα and IL-6 in the liver and in epididymal fat [99]. Recently, the anti-inflammatory effect of 7-HMR at a dose of 10 mg/kg has been demonstrated in an animal model of Parkinson’s disease, where this lignan has been shown to decrease the degeneration progression of striatal dopaminergic terminals and to improve motor performance, probably by means of a estrogen receptor beta (ERβ) mediated mechanism. In this model, a moderate decrease of mRNAs of pro-inflammatory mediators such as inducible nitric oxide synthetase (iNOS) and TNFα, and an increase of mRNAs of anti-inflammatory proteins such as transforming growth factor beta (TGFβ) and macrophage mannose receptor (CD206), were found [100].

7-HMR has estrogenic activity and can be found in the phytoestrogen family. Its estrogenic activity is slightly lower than estradiol (E2), showing effects in the nM–μM range. The estrogen receptor antagonist tamoxifene significantly reduces 7-HMR estrogenic effect in competition experiments [101].

The intestine is the first and the natural target for the action of lignans, because they are usually present in food and they can carry out all their antioxidant and anti-inflammatory potential during the absorption in enterocytes. In order to verify the potential effect of 7-HMR on iron metabolism in bowel cells, we used Caco-2 cell as a cellular model. Here we show preliminary results demonstrating the effects of 7-HMR on HEPC expression in this cellular model and discuss 7-HMR potential as nutraceutical against anemia related to CeD.

## 5. Experimental Results and Discussion

### 5.1. Anti-Inflammatory Effect of 7-HMR

Inflammation, determined by leucocytes that infiltrate bowel mucosa, plays a determinant role in CeD and other bowel diseases. Ingestion of foods enriched with anti-inflammatory nutraceutical molecules could ameliorate inflammation in these pathologies. Inflammation mediated by IL-6 production is strongly related to iron metabolism at two levels: a) IL-6, present in blood circulation, upregulates HEPC production by the liver at the systemic level, regulating systemic iron distribution and causing anemia of inflammation; b) the production of HEPC in tissues different from the liver has been shown as a consequence of local inflammatory stimuli [57]. In the second case, HEPC could induce iron compartmentalization mainly confined in these districts.

In CeD, the inflammatory stimulation caused by toxic peptides of gliadin or by IL-6 produced by immune cells might determine increased expression and secretion of HEPC in the small intestine that might induce ferroportin degradation through an autocrine–paracrine mechanism. This might determine the increase of cellular iron and the decrease of iron efflux. The block of iron export, caused by local inflammation, might exacerbate defective iron absorption due to reduced absorptive surface area in the intestine of patients presenting with anemia. 7-HMR could ameliorate this vicious circle by its anti-inflammatory effect, improving the local inflammatory insult and consequently contributing to ameliorate iron absorption.

**Figure 3:**
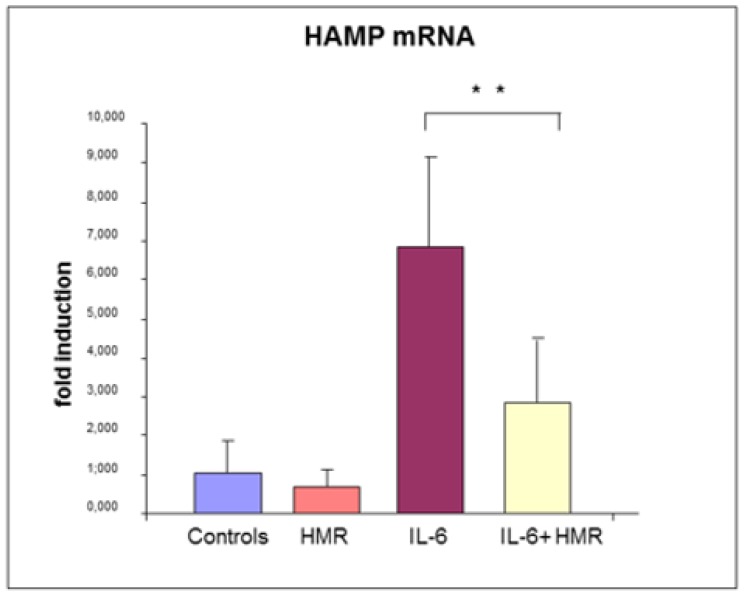
Caco-2 cells were treated with 50 ng/mL IL-6, in the presence or absence of 1μM HMR. Hepcidin mRNA expression was measured as fold induction by real time RT-PCR, compared to untreated cells. The results, are expressed as mean ± SD of three experiments [102]. ** *p* value < 0.001.

To verify the anti-inflammatory effect of 7-HMR in an in vitro model of bowel, Caco-2 cells were treated with IL-6 in the presence or absence of 7-HMR. The expression of HAMP mRNA after 24 h was analyzed as reported in supplementary materials (Figure 3).

7-HMR alone did not have significant effect on HAMP expression. HAMP mRNA was upregulated (*p* value < 0.01) by IL-6 treatment, as expected, while co-treatment with 7-HMR downregulated its expression, although without restoring basal levels of expression, at the dose and time studied. 

These data confirm that Caco-2 cells can induce HAMP under inflammatory stimulation, as previously reported [103,104], and that IL-6 significantly increases HAMP mRNA expression in these cells. The same effect could occur in bowel mucosa as a consequence of local inflammatory stimulation. HEPC local production could determine iron retention in enterocytes by means of ferroportin degradation or decreased functionality, causing the decrease of iron release in blood circulation, the reduction of TF saturation and ultimately iron deficiency anemia. Interestingly, 7-HMR decreases HAMP mRNA expression, suggesting its ability to reduce IL-6 effect, possibly by means of its anti-inflammatory effect. The decrease of HAMP mRNA expression could ameliorate iron absorption in vivo, increasing sideremia and TF saturation, thus mitigating anemia caused by flattened mucosa and consequent malabsorption [99].

### 5.2. Effect on Hepcidin Promoter

After transfection, Caco-2 cells were treated with 50 ng/mL IL-6 in presence or in absence of 1μM 7-HMR for 24 h to simulate the effect of inflammation. 7-HMR could exert its anti-inflammatory effect by inhibiting the activity of IL-6 at any point of the signal transduction pathway from activated receptors to HAMP promoter. To verify if the decrease of HAMP expression was due to an action on its promoter, Caco-2 cells were transiently transfected with pGL2-HAMP-LUC. This plasmid contains the reporter gene luciferase under the control of the region comprised between the bases −2900 + 1 of the HAMP 5′ flanking region [105].

**Figure 4:**
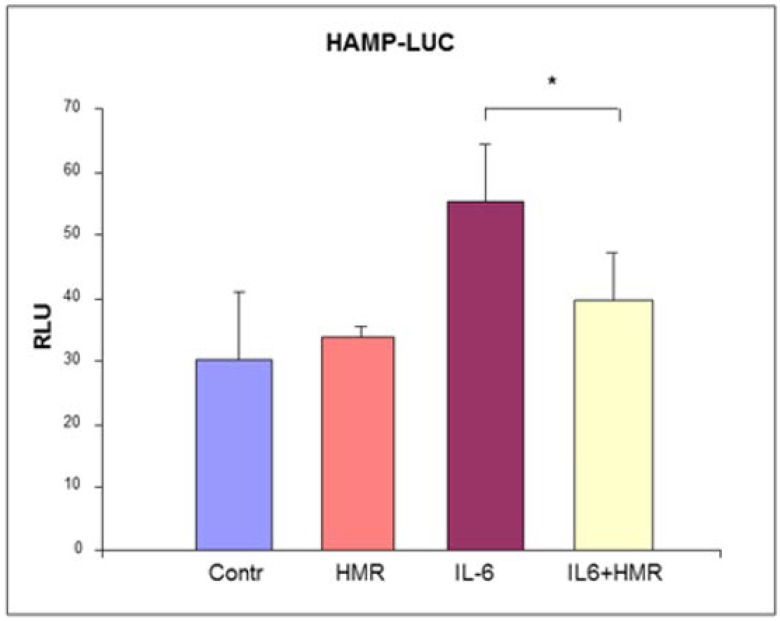
Caco-2 cells were transfected with pGL2-HAMP-LUC plasmid and treated with 50 ng/mL IL-6 in the presence or absence of 1 μM HMR. HAMP promoter activity was measured as Relative Luciferase Units (Firefly luciferase units, normalized with Renilla luciferase units), in comparison with untreated cells. The results are presented as mean ± SD of three experimets. * *p* value < 0.05

7-HMR alone did not have a significant effect on HAMP promoter. We observed an increase of the luciferase production by IL-6 treatment as expected, while 7-HMR significantly reduced this upregulation (*p* value < 0.05, the methods are described in supplementary materials) (Figure 4).

These data confirmed the functionality in Caco-2 cells of the STAT3 binding site regulatory sequence comprised between −72 and −64 in the HAMP promoter [64,106] and targeted by the IL-6 pathway. 7-HMR decreased the effect of IL-6 on the reporter gene expression in transfected cells, but the mechanism of this inhibition remains to be clarified. 7-HMR could interfere with the IL-6 induced pathway in any phase of the signal transduction. Recently, we showed the anti-inflammatory effect of 7-HMR through the downregulation of IL-6 mRNA level in epidydimal fat and liver of C57BJ/6 mice on a high-fat diet [99]. The results presented here showed the effect of 7-HMR on the HAMP promoter, which was able to counteract the upregulation of IL-6. 

It has been reported that estrogen decreases HAMP expression through the ERE half site located between −2474 and −2462 [59] and/or through a second ERE found at −1244 to −1232 of HAMP promoter [60]. These regions are comprised in the promoter region cloned in the pGL2-HAMP-LUC plasmid. It has been reported that 7-HMR could exert estrogenic activity by means of estrogen receptor pathways with the same mechanism used by E2 [101]. Therefore, it is tempting to speculate that 7-HMR could be able to counteract the IL-6 HAMP upregulation through its inhibitory estrogenic activity. Alternatively, 7-HMR could both reduce expression of IL-6, as shown in an animal model [99], and attenuate the inflammatory effect of IL-6 by acting as an estrogen-like molecule through an effect that target HAMP promoter.

The here reported observations are only preliminary data and more definitive data could be obtained through more detailed dose response and time course studies. The effects of IL-6 and 7-HMR and their combination on other iron-related proteins, such as ferroportin, DMT1, FT and TFR1 in Caco-2 cells, could add important information about iron metabolism in Caco-2 cells as a model of gut. However, considering that HEPC is the master regulator of iron metabolism, the possibility that 7-HMR could downregulate IL-6 effects on HAMP promoter is of importance. This observation allows us to speculate that lignans intake (in particular 7-HMR) by patients affected by CeD or other bowel diseases could help to ameliorate iron absorption by limiting the effects of local inflammation, both by anti-inflammatory and estrogenic activity.

In conclusion, immune cells infiltrate the intestinal mucosa producing inflammatory cytokines such as IL-6, TNF-α and IL-1β in CeD and other IBDs. These inflammatory cytokines could increase local HEPC expression, decreasing iron absorption and worsening patients’ anemia. Our preliminary study shows that the lignan 7-HMR significantly decreased IL-6-induced hepcidin mRNA expression in Caco-2 cells. Furthermore, 7-HMR was effective in decreasing the transcription of HAMP gene in the presence of IL-6. Although this evidence needs further studies, it confirms that 7-HMR is a lignan characterized by very effective nutraceutical properties.

## Figures and Tables

**Table 1:** Concentration of 7-Hydroxymatairesinol in common nutrient sources. The table presents the concentration of 7-HMR (μg/100 g) in common foods. Gluten free foods are shown in italics.

Nutrient Sources	7-Hydroxymatairesinol (μg/100 g)
*Amaranth*	519
Barley bran	541
*Buckwheat*	142
*Corn*	407
*Flaxseed*	35
*Millet*	160
Oat bran	712
*Quinoa*	163
*Rye Bran*	1017
*Sesame*	7209
Wheat Bran	2787

## Supplementary Materials

Material and Methods are explained in supplementary materials.
