# Supplementary material for: Iron Absorption in Celiac Disease and Nutraceutical Effect of 7-Hydroxymatairesinol. Mini-Review
Source: Molecules. 2020 Apr 27;25(9):2041. doi: 10.3390/molecules25092041 (PMC7249079; doi:10.3390/molecules25092041)
Supplement: Supplementary file 1 [file molecules-25-02041-s001.pdf]

Supplementary Materials

1. Materials and Methods

1.1. Chemicals

7-HMR ((−)-Hydroxymatairesinol, mixture of epimers) was bought from Sigma-Aldrich. IL-6 was purchased from ReliaTech (Receptor Ligand Technologies GmbH).

1.2. Cells Culture and treatment.

Caco-2 Cell line was purchased by IZSLER Brescia and was cultured in Dulbecco's Modified Eagle's Medium (DMEM) (Gibco) added with 10 % fetal valf serum (Gibco), flutamine 1mM (Lonza) and 100X penicillin (10000 U)/streptomycin 10mg/ml (Sigma). Cells were grown at 37°C in humidified air with 5% CO_2.

Confluent Caco-2 were respectively treated with 50 ng/ml IL-6 or 50 ng/ml IL-6 plus 1 μM 7-HMR or only 1μM 7-HMR. After 24h total, RNA was extracted with TriReagent Solution (Ambion). Genomic DNA was digested using DNAseI (Promega) and reverse-transcribed using Im-PromII Reverse Trancriptase (Promega) to obtain cDNA, as described in Zanella et al. 2008 [102]. HAMP mRNA was analysed by quantitative RT-PCR using Assay on Demand kits (Applied Biosystem) on an ABI PRISM 7000 (Applied Biosystem). Hypoxantine phosphorybosiltransferase 1(HPRT1) mRNA was used to normalize HAMP expression by the relative quantification method ($2^{-\Delta\Delta Ct}$) as previously described [72].

Caco-2 cells at 80%–90% of confluence were co-transfected with 1 μg HAMP promoter-LUC plasmid (pGL2-HAMP-Luciferase) kindly provided by Prof. Camaschella [105] and 100ng of Renilla luciferase plasmid (Promega) using Lipofectamine 2000 reagent (Thermo Fischer Scientific), as recommended by the manufacturer's instructions. After 24h, cells were treated respectively with 50 ng/ml IL-6, 50 ng/ml IL-6 plus 1 μM 7-HMR and with only 1μM 7-HMR. After 24 hours, cells were harvested and lysates were analysed, using the Dual-Glo Luciferase Assay System (Promega), as recommended by manufacturer's instructions. The photon emissions of extracted cells were quantified using a luminometer (Centro LB 960, Berthold Technologies).

1.3. Statistical Analysis

For each test, three independent experiments were performed. mRNA expression and luciferase activity were expressed as fold induction and relative RLU respectively. Statistical analysis was performed using two-way T-Student analysis of variance test.
